# Editorial

**Published:** 1896-02

**Authors:** 


### Editorial.

It is our melancholy task to chronicle the death of two of the most conspicuous members of the medical profession in the South, Dr. Thomas S. Powell, of Atlanta, and Dr. James E. Reeves, of Chattanooga.

Dr. Thomas Spencer Powell died at his residence in Atlanta, December 27, 1895, of cerebral apoplexy. We regret that space permits us to give only a brief sketch of this distinguished man.

Dr. Powell was born at Petersburg, Virginia. He received his classical education in the schools of Brunswick Co., Virginia. Coming to Georgia at an early age, he was associated with the profession in this state for over forty years. Dr. Powell was a man of indefatigable energy and great decision of character and was constantly active in devising plans for the promotion and welfare of the profession. In 1870, he founded the Southern Medical Record, and though long since disconnected with it, he was ever its friend and well-wisher. Dr. Powell first exhibited, in the columns of this journal, his distinguished ability as a writer, and gave evidence of his high professional and scientific attainments.

As founder and executive head of the Southern Medical College, Dr. Powell was widely known, and much of the success of this famous institution was due to his wise administration of its affairs. Of late years, his interest in this institution may be justly regarded as the absorbing motive of his life.

Dr. Powell was an avowed enemy of fraud and quackery, and displayed great activity in helping to secure the passage of the law regulating the practice of medicine and surgery in Georgia. He had the highest regard for the sanctity of the Hippocratic oath, and regulated his whole life by the principle that physician and gentleman are synonymous terms. Dr. Powell was in the truest sense a public benefactor and philanthropist, and his faithful services to his fellow-men are destined to survive him in the gratitude and appreciation of the people of this state.

Dr. James E. Reeves died at Chattanooga, Tennessee, January 4, 1896, of what was supposed to be cancer of the liver. Dr. Reeves was born in Rappahannock Co., Virginia, 1829. He graduated from the University of Pennsylvania in 1860. He lived for some years in Wheeling, West Virginia, and then removed to Chattanooga, Tennessee, where he resided until his death.

Dr. Reeves was one of the founders of the American Public Health Association, and was its president in 1885. His works in microscopy and hygiene are well known. His contributions to the medical press were numerous and covered a wide range of subjects. About a year ago, he published a small work on the use of the microscope. Dr. Reeves was a gentleman of the old regime, and his death will be a grievous loss to many friends in and out of the profession.

DR. W. A. CROW, adjunct professor] of obstetrics at the Southern Medical College, has been appointed to fill the chair, as full professor, made vacant by the death of Dr. Powell. Dr. Crow is admirably fitted to fill the chair, and his selection will, without doubt, reflect great credit upon the college.

THE *St. Louis Medical Fortnightly* has issued a small brochure, containing half-tone portraits of its editorial staff. We notice among these, twenty-five uncommonly handsome and intelligent-looking medical gentlemen, not less than eight of whom affect side-whiskers, varying in extent from the firm, short-cropped "slugger" to the breezy Dundreary of other days. It would be of interest to know if this is a mere local affectation in St. Louis, or comes from an exuberant development of that region of the face from which such whiskers spring.

NEW MEDICAL JOURNALS.—The following fledgelings have been hatched out with the New Year: *Langsdale's Lancet*, *Peoria Medical Journal* (resuscitated), *Cleveland Medical Journal;* relic of the late lamented *Western Reserve Medical Journal Southwestern Medical and Surgical Reporter*, late *Hygeia*, of Fort Worth, Texas.

THE attention of him who anticipates fathering a new medical journal, is called to the fact that the names "Record," "Journal," "Lancet," "Reporter," and the like, have been appropriated and that there is absolutely no room for another. Appropriate titles are not lacking, however, so long as there is not yet a "Medical Perforator" or "Hypnotic" or a "Depressant." The "Monthly Anti-Scorbutic" would be an apt title for some periodical, whose mission it was to furnish fresh food instead of the stale stuff of foreign importation which is dished out to long-suffering subscribers by so many American medical journals.

---

P. BLAKISTON, SON & CO., of Philadelphia, announce a book on "Appendicitis," by John B. Deavor, M.D., assistant professor of applied anatomy, University of Pennsylvania; assistant surgeon to the German hospital, etc. The book will be arranged in a practical and systematic manner. The history, ætiology, symptoms, diagnosis, operative treatment, prognosis, and complications of this disease will be given in the order named. It will contain about forty illustrations of methods of procedure in operating, and typical pathological conditions of the appendix, the latter being printed in colors.

---

*The Virginia Medical Monthly*, for so long the representative medical journal of the South, will be issued after April as a semimonthly. Under the able management of our old friend, Dr. Landon B. Edwards, this journal has enjoyed a prosperity and has achieved a reputation that must be a source of gratification to him as it is to his friends.

We wish for the semimonthly the same success and usefulness that have marked the course of the parent publication.

